# The Asymmetric Threat of Maternal Cell Contamination in Prenatal Single-Gene Testing

**DOI:** 10.3390/diagnostics16152302

**Published:** 2026-07-23

**Authors:** Mengmeng Li, Jieping Song, Kui Sun, Jiazhen Chang, Kaili Yin, Xueting Yang, Yan Lv, Yulin Jiang, Na Hao

**Affiliations:** 1 National Clinical Research Center for Women’s Health and Obstetric and Gynecologic Diseases, Department of Obstetrics and Gynecology, Peking Union Medical College Hospital, Chinese Academy of Medical Sciences & Peking Union Medical College, Beijing 100730, China; limengmeng@pumch.cn (M.L.); changjiazhen@pumch.cn (J.C.); yinkaili@pumch.cn (K.Y.); yangxueting@pumch.cn (X.Y.); lvyan@pumch.cn (Y.L.); 2 Medical Genetics Center, Maternal and Child Health Hospital of Hubei Province, Wuhan 430070, China; songjieping@hbfy.com; 3 Prenatal Diagnosis Center, The Second Affiliated Hospital of Bengbu Medical University, Bengbu 233000, China; kui0113@163.com

**Keywords:** maternal cell contamination, whole exome sequencing, Sanger sequencing, risk stratification, prenatal single-gene testing

## Abstract

**Background/Objectives:** Maternal cell contamination (MCC) is a pervasive challenge in prenatal single-gene testing, as it can cause both false-positive and false-negative results. Despite its clinical significance, quantitative tolerance thresholds for MCC in whole exome sequencing (WES) and Sanger sequencing remain limited. **Methods:** We established a gradient contamination model (5–95%) using genomic DNA from 20 mother–child pairs and assessed the detection fidelity for single-nucleotide variants (SNVs) and insertions/deletions (InDels) in two clinically relevant scenarios using both WES and Sanger sequencing—Scenario 1 (Fetus Heterozygous/Mother Wild-type) and Scenario 2 (Fetus Wild-type/Mother Heterozygous). **Results:** In Scenario 1, analysis of the 20 loci evaluated by both WES and Sanger sequencing revealed false-negative rates of 0% when MCC ≤ 30%, which then increased sharply across the 30–70% MCC range and reached 100% at 95% MCC. In Scenario 2, analysis of 12,627 WES loci showed a false-positive rate of ≤1.0% when MCC ≤ 10%, which then increased from 1.0% to 76.6% within the 10–30% MCC range and climbed to 89.8% at 50% MCC. A similar pattern was observed for the 20 loci analyzed by both WES and Sanger sequencing in this scenario. These findings potentially support a three-tier stratification: low-risk (MCC ≤ 30% in Scenario 1 and ≤10% in Scenario 2), moderate-risk (30% < MCC < 70% in Scenario 1, 10% < MCC < 30% in Scenario 2), and high-risk (MCC ≥ 70% in Scenario 1 and ≥30% in Scenario 2). **Conclusions:** Our findings reveal a clear directional asymmetry of MCC tolerance between the two scenarios, indicating that MCC tolerance depends not only on contamination level but also on the genotype of the contaminating DNA. We further propose a clinical reference framework based on a three-tier risk stratification for prenatal single-gene testing, which can guide result interpretation and laboratory decision-making, including decisions about reliable reporting, orthogonal validation, or re-sampling.

## 1. Introduction

Maternal cell contamination (MCC) refers to the unintended presence of maternal cells in fetal samples, predominantly occurring in invasive prenatal procedures such as chorionic villus sampling (CVS) and amniocentesis [1]. MCC represents a quality control challenge that undermines the precision of prenatal genetic testing [1]. By diluting fetal genetic signals, MCC obscures the correct interpretation of inheritance patterns and leads to incorrect fetal genotypes, manifesting as false negatives or false positives [2,3]. A false-negative finding may misclassify an affected fetus as unaffected, thereby removing the opportunity for informed decision-making and timely intervention. A false-positive outcome may lead to unnecessary termination of a healthy pregnancy. Consequently, establishing quantitative tolerance thresholds for MCC across different diagnostic methods is essential for reliable prenatal diagnosis.

MCC arises primarily from the admixture of maternal decidual tissue or peripheral blood cells. Its prevalence varies with both sample type and collection technique. The estimated risk of MCC is approximately 0.5% for amniotic fluid, 1–2% for CVS, and 4% for umbilical cord blood [4,5,6]. Uncultured amniocytes have a higher risk of MCC than cultured ones, owing to maternal blood contamination in the cell pellet [7]; in contrast, cultured CVS carries a substantially greater risk of MCC from residual maternal decidua compared to uncultured CVS [8]. Furthermore, MCC levels exhibit considerable heterogeneity. In a study of celomic fluid samples collected during 7–9 weeks of gestation, approximately 33.1% showed minimal contamination (<5%), 53% exhibited moderate contamination (5–60%), and 13.9% exceeded 60% [9]. This wide distribution underscores the need for empirically defined tolerance thresholds to guide clinical interpretation.

MCC is commonly quantified using short tandem repeat (STR) analysis, which estimates contamination by analyzing peak pattern shifts across multiple highly polymorphic loci [4,10]. Other methods include single-nucleotide polymorphism (SNP) arrays [11], chip-based digital PCR techniques [12], and amplicon sequencing or methylation-sensitive assays [13]. More recently, methods have been developed to estimate contamination directly from whole exome sequencing (WES) data [14].

MCC affects multiple prenatal diagnostic methods, including karyotyping [2], quantitative fluorescent PCR [15], and chromosomal microarray analysis [16]. With the growing adoption of prenatal molecular diagnostics for monogenic disorders, WES [17] and Sanger sequencing [18] have become standard tools for detecting Mendelian disease variants; nonetheless, both remain susceptible to MCC interference [19]. Prior investigations have demonstrated that even 5% MCC can cause false-negative results in Sanger sequencing, while approximately 30% MCC can completely prevent detection of pathogenic variants [20]. WES is similarly affected [19]. Moreover, WES data analysis requires rigorous quality control and variant filtering [17,21], and MCC further complicates variant calling and interpretation [14,19,22].

The impact of MCC on prenatal diagnosis depends on the clinical scenario. In families with a molecularly characterized proband in which the mother harbors a wild-type genotype, prenatal testing primarily seeks to ascertain whether the mutation detected in the fetus is de novo or paternally inherited. In this context, the main risk is false negatives, since maternal wild-type DNA dilutes the fetal mutant allele (hereafter, Scenario 1: Fetus Heterozygous/Mother Wild-type). Conversely, when the mother is a known carrier of a pathogenic mutation, the main risk is false positives, as the maternal heterozygous signal is erroneously attributed to the fetus (hereafter, Scenario 2: Fetus Wild-type/Mother Heterozygous).

These two scenarios represent most clinical indications for prenatal single-gene testing for monogenic disorders, but their tolerance to MCC differs. To date, a systematic, quantitative evaluation delineating the tolerance thresholds of WES and Sanger sequencing for single-nucleotide variants (SNVs) and insertions/deletions (InDels) within these scenarios remains lacking. This gap leaves laboratories with a difficult choice when faced with contaminated samples: recommend re-sampling (with miscarriage risk) or issue a diagnostic report that may carry a false-positive risk. To address this, we systematically assessed WES and Sanger sequencing performance across a gradient of MCC levels using a paired mother–child design. Our objective is to provide quantitative reference standards to define empirical boundaries for MCC risk stratification and result interpretation, thereby assisting laboratories in determining when a contaminated sample can be reliably reported and when additional measures (such as orthogonal validation or re-sampling) are warranted.

## 2. Materials and Methods

### 2.1. Sample Collection and MCC Sample Preparation

This investigation received formal approval from the Ethics Review Committee of Peking Union Medical College Hospital, Chinese Academy of Medical Sciences (Approval No.: I-24PJ2648). Due to the retrospective nature of this study, informed consent was waived. All procedures involving human subjects were conducted in strict adherence to the ethical standards enshrined in the Declaration of Helsinki.

The study retrospectively enrolled paired maternal and offspring samples confirmed via prenatal diagnosis, encompassing fetal specimens harboring de novo SNVs or InDels pathogenic for single-gene disorders, alongside their maternal counterparts. Peripheral blood samples from both mothers and neonates were collected, with genomic DNA extracted following standardized protocols and stored at −20 °C until further use.

Quantification of fetal and maternal genomic DNA was performed using a Qubit 4 fluorometer (Thermo Fisher Scientific, Waltham, MA, USA) in conjunction with the Qubit dsDNA High Sensitivity (HS) Assay Kit (Thermo Fisher Scientific, Waltham, MA, USA). Subsequent normalization of each DNA sample set to equimolar concentrations was achieved using nuclease-free water to establish stock solutions for the construction of defined contamination gradients. Utilizing fetal DNA as the baseline matrix, maternal DNA was incrementally introduced at predetermined mass fractions. Rigorous homogenization yielded gradient samples simulating varying degrees of MCC. The proportion of MCC was systematically varied at the following intervals: 5%, 10%, 15%, 20%, 30%, 50%, 60%, 70%, 80%, 90%, and 95%.

### 2.2. Assessment of MCC Impact on WES

Whole exome capture was executed utilizing the Twist Exome 2.0 panel (Twist Bioscience, San Francisco, CA, USA), with library preparation performed via the Watchmaker DNA Library Prep Kit with Fragmentation (Watchmaker Genomics, Boulder, CO, USA) in accordance with manufacturer protocols. Library quantification was performed using the Qubit dsDNA HS Assay Kit (Thermo Fisher Scientific, Waltham, MA, USA), and library fragment size distribution was evaluated using an Agilent 2100 Bioanalyzer (Agilent Technologies, Santa Clara, CA, USA). Libraries meeting quality criteria were sequenced on a NextSeq 550AR instrument (Annoroad Gene Technology, Beijing, China), employing 150 base pair paired-end reads, achieving an average depth of 100×, Q30 scores exceeding 0.85, and coverage exceeding 95% at 30× depth.

Raw sequencing reads were processed using fastp (v1.0.1) for quality control. Reads with more than 50% bases below Q20, more than five ambiguous bases (*N*), or a length shorter than 50 bp were removed, and Illumina paired-end adapter sequences were automatically trimmed. Clean reads were aligned to the human reference genome (GRCh37, hs37d5) using BWA-MEM (v0.7.17-r1188) with a minimum seed length of 32 (−k 32) and six computational threads while retaining read group information. Sequencing quality was evaluated by average sequencing depth, target coverage, and Q30 metrics. Germline variants were identified using GATK HaplotypeCaller (version 4.2.0.0) following the GATK Best Practices workflow. HaplotypeCaller was run with default parameters without additional optimization.

For the purpose of this study, SNV and InDel loci with sequencing depth > 30× and VAF ≥ 15% were classified as heterozygous to provide a standardized criterion for comparing variant detectability across different MCC levels. This threshold was applied solely for the experimental evaluation of MCC effects and does not replace comprehensive clinical variant interpretation, which also incorporates multiple quality metrics and caller-specific confidence models.

To assess the performance under varying MCC burdens, WES was performed on each contamination gradient sample. For Scenario 1 (Fetus Heterozygous/Mother Wild-type), corresponding loci across 20 mother–child pairs were interrogated to assess the influence of MCC on fetal variant detection sensitivity. For Scenario 2 (Fetus Wild-type/Mother Heterozygous), loci where the maternal genotype was heterozygous (VAF between 0.4 and 0.6) and the fetal genotype was wild-type (VAF = 0) were selected to evaluate MCC-induced interpretative confounders.

### 2.3. Assessment of MCC Impact on Sanger Sequencing

Targeted PCR primers were meticulously designed to amplify regions encompassing the variants of interest. Following PCR amplification and product purification, Sanger sequencing was conducted using an ABI 3730XL genetic analyzer (Thermo Fisher Scientific, Waltham, MA, USA). Variant detection thresholds were predicated upon the relative intensity of the mutant electropherogram peak: samples exhibiting a mutant peak height ≥ 20% of the corresponding wild-type peak were designated heterozygous, whereas those with mutant peak heights below this threshold were classified as wild-type.

Sanger sequencing assays were conducted across all contamination gradient samples to evaluate the effects of MCC on fetal variant detection under Scenario 1 (Fetus Heterozygous/Mother Wild-type). Concurrently, using data derived from WES, we filtered loci within coding exonic regions where mothers were heterozygous and fetuses were wild-type, in order to determine the degree of MCC interference on diagnostic interpretation under Scenario 2 (Fetus Wild-type/Mother Heterozygous).

### 2.4. Statistical Analysis

Comparisons of VAF distributions between SNVs and InDels at each MCC gradient were performed using the Mann–Whitney U test (two-tailed) with FDR correction (two-stage step-up method of Benjamini, Krieger, and Yekutieli). For comparisons between WES and Sanger sequencing performance—both applied to the identical set of loci—McNemar’s test was used for paired binary outcomes (correct versus incorrect genotyping calls) at each contamination level. All statistical tests were two-sided, and a *p* < 0.05 was considered statistically significant. Analyses were performed using GraphPad Prism 8.0.

## 3. Results

### 3.1. MCC Interference Effects in Scenario 1 (Fetus Heterozygous/Mother Wild-Type)

Twenty loci (10 SNVs and 10 InDels) were distributed across autosomes and the X chromosome, including missense, nonsense, conserved in-frame insertions, and frameshift mutations (Table S1). These loci resided in genes associated with monogenic disorders, such as MECP2, FBN1, TSC1, SCN9A, and FOXG1.

WES outcomes for SNV and InDel loci are detailed in Table S2, with summary statistics summarized in Table 1. When MCC ≤ 30%, all 20 loci were correctly detected as heterozygous by WES. False negatives for InDels and SNVs first appeared at 50% MCC (10%, 1/10) and 60% MCC (20%, 2/10), respectively. At 70% MCC, false-negative rates increased to 60% (6/10) for SNVs and 80% (8/10) for InDels. Beyond 80% MCC, nearly all loci were classified as wild-type.

Although SNVs and InDels appeared to show broader VAF dispersion at certain MCC levels (e.g., 10%, 20%, and 50%), the Mann–Whitney U test did not reach statistical significance at any gradient (Table S3). This suggests that the central tendencies of the two variant types remained comparable, allowing them to be pooled for subsequent statistical analysis.

**Figure 1:**
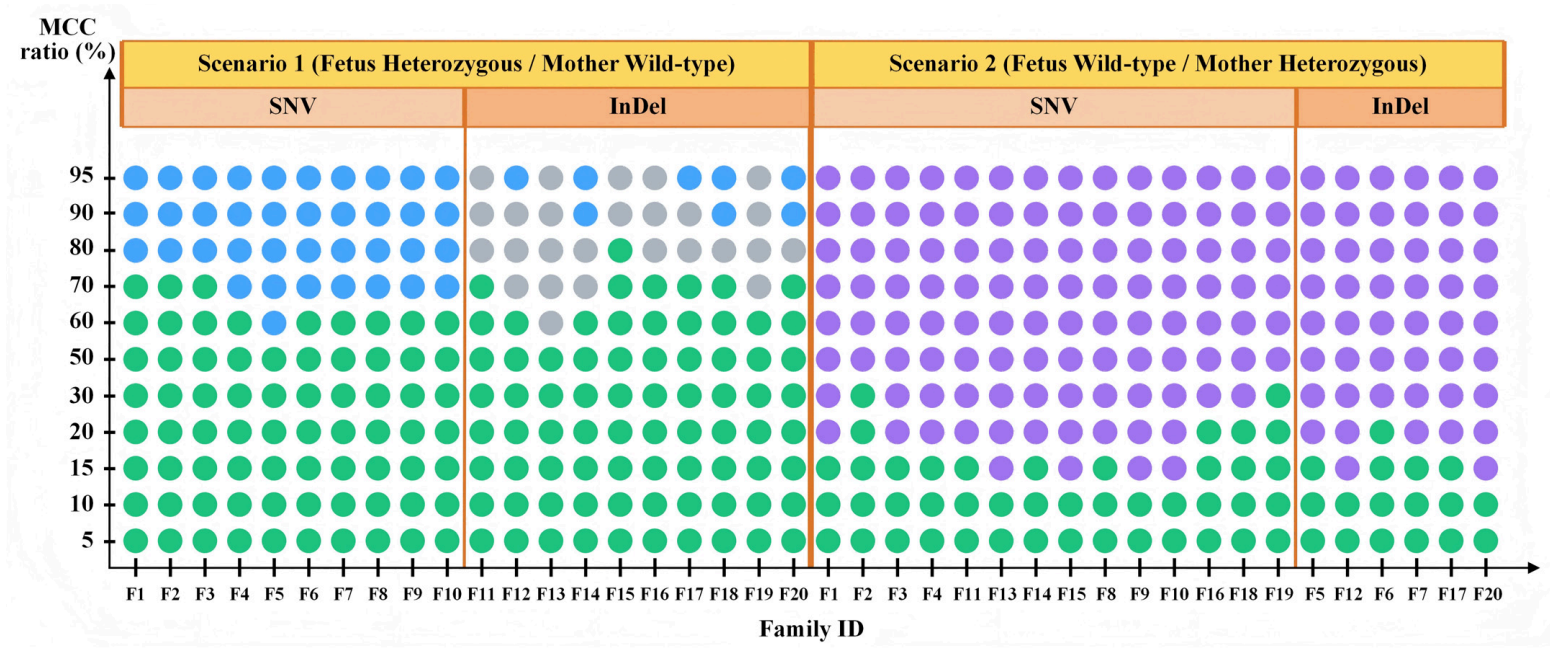
Heatmap of Sanger sequencing genotyping calls across MCC gradients and variant types in two clinical scenarios. Rows indicate MCC levels (5–95%); columns indicate 20 loci from 20 families. In Scenario 1 (Fetus Heterozygous/Mother Wild-type), the expected call is heterozygous. Green: correct heterozygous call; Blue: false-negative call; Gray: indeterminate call (secondary peaks present but below the 20% calling threshold). In Scenario 2 (Fetus Wild-type/Mother Heterozygous), the expected call is wild-type. Green: correct wild-type call; Violet: false-positive call.

The Sanger sequencing results are illustrated in Figure 1. When MCC ≤ 50%, all SNVs and InDels were correctly identified as heterozygous. At 60% MCC, one SNV locus was misidentified as wild-type, and one “indeterminate” InDel was also observed (Figure 1). This InDel showed clearly distinguishable double peaks, yet the mutant peak height fell below the 20% calling threshold. Accordingly, we labeled such InDels as “indeterminate” separately and discussed them later, though they were still counted as false negatives in statistical analyses. At 70% MCC, false negatives became frequent for both SNVs (70%, 7/10) and InDels (40%, 4/10). When MCC ≥ 80%, few loci were correctly identified.

From these results, Sanger sequencing appeared to be less susceptible to MCC than WES, but McNemar’s test on the same 20 loci showed no significant difference in detection accuracy between these two methods at any MCC gradient (exact binomial *p* > 0.05; Table S4), suggesting comparable susceptibility.

### 3.2. MCC Interference Effects in Scenario 2 (Fetus Wild-Type/Mother Heterozygous)

WES data from 12,627 variants showed a false-positive rate of 0.2% at 5% MCC and 1.0% at 10% MCC, followed by a sharp increase to 12.6% at 15% MCC, 40.4% at 20% MCC, and 76.6% at 30% MCC. At 50% MCC, the false-positive rate reached 89.8% (Table 2).

Given that the above findings for 12,627 loci were based solely on bioinformatic analysis, we chose 20 of these loci (including 14 SNVs and 6 InDels, Table S1) for direct statistical comparisons between WES and Sanger sequencing (Table 1). At 5% and 10% MCC, all 20 loci were correctly identified. At 15% MCC, all 14 SNVs remained correct, whereas two InDels (33.3%) were misidentified as heterozygous. At 20% MCC, false-positive rates reached 57.1% (8/14) for SNVs and 33.3% (2/6) for InDels. At 30% MCC, these rates increased to 71.4% (10/14) and 66.7% (4/6), respectively. When MCC ≥ 50%, nearly all loci were misidentified.

Mann–Whitney U tests showed no significant difference between SNVs and InDels at any gradient in this scenario (Table S3), indicating that both mutation types follow similar trends.

In Sanger sequencing of the same 20 loci, when MCC ≤ 10%, all loci were correctly identified; at 15% MCC, false positives emerged in both SNVs (28.6%, 4/14) and InDels (33.3%, 2/6); at 20% MCC, false-positive rates rose sharply to 71.4% (10/14) for SNVs and 83.3% (5/6) for InDels. When MCC ≥ 30%, nearly all loci were false positives (Figure 1).

Like the statistical results in Scenario 1, there was also no significant difference between WES and Sanger sequencing in detection accuracy at any MCC gradient in Scenario 2 (McNemar’s test, exact binomial *p* > 0.05; Table S4).

### 3.3. Integrated Comparison of the Two Scenarios

Taken together, the impact of MCC on WES and Sanger sequencing shows significant context-dependency, and the data potentially support a three-tier risk stratification into low-, moderate-, and high-risk zones (Table 3).

The boundaries between the three zones were selected based on the observed error patterns: the boundary between low- and moderate-risk zones is set at the maximum MCC level where the false-negative rate is 0% across all 20 loci in Scenario 1, or where the false-positive rate falls below 1.0% across 12,627 loci in Scenario 2. The boundary between the moderate- and high-risk zones is defined at the minimum MCC level where either the false-negative or false-positive rate exceeds 50%.

Consequently, in Scenario 1, MCC ≤ 30% defines the low-risk zone; 30% < MCC < 70% defines the moderate-risk zone; and MCC ≥ 70% defines the high-risk zone. In Scenario 2, MCC ≤ 10% defines the low-risk zone; 10% < MCC < 30% defines the moderate-risk zone; and MCC ≥ 30% defines the high-risk zone.

## 4. Discussion

### 4.1. Asymmetric MCC Tolerance in Prenatal Single-Gene Testing

MCC represents an inherent technical challenge in prenatal molecular diagnostics. Although its clinical impact is widely recognized, systematic comparisons of MCC tolerance across different detection methods remain limited. The present study provides empirical estimates of MCC impact on SNV and InDel detection via WES and Sanger sequencing using a paired mother–child gradient contamination model. Our findings suggest a directional asymmetry in MCC tolerance between Scenario 1 (Fetus Heterozygous/Mother Wild-type) and Scenario 2 (Fetus Wild-type/Mother Heterozygous), with potential implications for quality control, result interpretation, and genetic counseling.

This asymmetry reflects a fundamental difference in how contamination affects signal interpretation. In Scenario 1, the fetal variant signal is progressively diluted by wild-type maternal DNA. As a result, the low-risk zone extends to 30% MCC, with no false negatives observed across the 20 loci examined. In the moderate-risk zone (30% < MCC < 70%), the false-negative rate increases sharply with contamination, reaching approximately 70% at the upper boundary. In the high-risk zone (MCC ≥ 70%), misidentification becomes nearly complete. In Scenario 2, the contaminating maternal DNA carries the variant allele itself, which actively introduces a false-positive signal into a background that should be wild-type. As a result, the low-risk zone is much narrower, extending only to 10% MCC, where the false-positive rate is ≤1.0% in the large-scale WES dataset (12,627 loci) and 0% in the 20 shared loci. In the moderate-risk zone (10% < MCC < 30%), false-positive rates rise sharply, from near-zero to over 70%, while in the high-risk zone (MCC ≥ 30%), the rates approximate 100%.

This directional asymmetry carries a clear clinical message: no single universal MCC cutoff applies across all prenatal diagnostic scenarios. Laboratories must instead tailor their interpretation strategies to the specific genetic context, distinguishing between cases where the mother is wild-type and those where she is a known carrier. In Scenario 1, the broader low-risk zone permits generally reliable reporting at MCC levels of ≤30%, whereas in Scenario 2, the narrower low-risk zone necessitates stricter quality control at MCC levels of ≤10%. Orthogonal validation or re-sampling is advised for samples falling within the moderate- or high-risk zones.

Our findings also align with and extend the observations of Koczok et al. [20], who evaluated Sanger sequencing, pyrosequencing, and MLPA under genetic conditions identical to our Scenario 2 (Fetus Wild-type/Mother Heterozygous). They found that Sanger sequencing tolerated up to 5% to 30% MCC before the fetal genotype became ambiguous, while pyrosequencing was markedly more sensitive (detecting as low as 1% MCC), and MLPA yielded diagnostic uncertainty only at MCC ≥ 40%. Notably, they also predicted in their discussion that the MCC tolerance would be substantially higher if the contamination direction were reversed, with a wild-type mother and a fetus carrying a paternal variant (corresponding to our Scenario 1). Our data support this prediction and further show that the asymmetry persists across both WES and Sanger sequencing. Moreover, the heightened sensitivity of pyrosequencing in their study parallels our observation in the large-scale WES dataset, where a residual false-positive rate (0.2% at 5% MCC, 1.0% at 10% MCC) persisted within the low-risk zone in Scenario 2. This parallel, observed across pyrosequencing and WES despite differences in sequencing chemistry, suggests that the directional asymmetry is unlikely to be platform-specific and may reflect a general feature of sequencing-based prenatal testing.

### 4.2. Broader Clinical Implications

From a clinical standpoint, our study provides a quantitative risk stratification for MCC management in prenatal single-gene testing. By distinguishing between low-, moderate-, and high-risk zones, this risk stratification offers laboratories a structured basis for deciding whether a contaminated sample can be interpreted with confidence or requires additional scrutiny, thereby potentially reducing unnecessary invasive procedures and guiding the appropriate use of orthogonal validation across both clinical scenarios.

Our findings also highlight the importance of maternal carrier screening. In the absence of maternal genotype information, a negative fetal result can be misattributed to maternal cell contamination (MCC), whereas identifying the mother as a carrier enables early detection of loci at elevated MCC risk. More broadly, this supports an integrated approach to prenatal genetic evaluation, in which carrier screening, prenatal diagnosis, and MCC assessment are coordinated—an objective that depends on collaboration among clinicians, laboratory personnel, and genetic counselors.

### 4.3. Practical Considerations for InDel Interpretation in Sanger Sequencing

In the Sanger sequencing analysis of Scenario 1, several InDel loci exhibited overlapping electropherogram peaks downstream of the mutation site, which we classified as “indeterminate” in Figure 1. Although they were treated as false negatives for statistical purposes, their trace appearance differed from that of clean wild-type sequences, warranting separate consideration.

Notably, the proportion of indeterminate calls among all false negatives decreased progressively with increasing MCC: from 100% (1/1) at 60% MCC to 50% (5/10) at 95% MCC (Figure 1). This pattern suggests that in the high-risk zone, Sanger sequencing may still detect discernible fetal DNA signals for InDels even when mutant peak heights fall below the 20% calling threshold. In other words, the false-negative rate may overestimate the true information loss at high MCC levels.

This phenomenon arises from two factors: (1) InDel loci often reside in repetitive regions, where PCR amplification is prone to polymerase slippage and stutter peaks [23]; (2) co-amplification of fetal and maternal alleles creates sequence length differences, leading to electrophoretic phase shifts and overlapping peaks [24]. The combination of these effects increases chromatogram complexity and complicates the application of a fixed 20% calling threshold.

Importantly, the observation of a decreasing indeterminate proportion with rising MCC is derived from a limited set of InDel loci (*n* = 10). Whether this pattern holds more broadly requires validation in larger, more diverse cohorts.

### 4.4. Strengths of This Study

This study possesses several methodological strengths. First, paired mother–child samples with confirmed kinship ensured a genetically isogenic background, reducing confounding from unrelated allelic heterogeneity. Second, the comprehensive gradient design allowed detailed mapping of the dose–response trajectory, uncovering distinct patterns of error increase that would be overlooked in coarse comparisons (e.g., “low” versus “high” contamination). Third, to our knowledge, this study provides the first systematic comparison of WES and Sanger sequencing specifically designed to capture the directional asymmetry of MCC interference, which directly reflects the two most common prenatal scenarios.

### 4.5. Limitations

This study has several limitations. First, although our artificially mixed DNA model helped define empirical boundaries, it does not fully capture the biological and technical heterogeneity of prenatal specimens, where variations in DNA quality, PCR inhibitors, or allelic dropout may exacerbate MCC interference. Second, WES outcomes are influenced by platform-specific variables (sequencing instrument, capture kit, depth, and bioinformatic pipeline). Therefore, our boundaries should not be regarded as universal clinical action thresholds and must be locally validated before clinical adoption. Third, the 20 loci examined in Scenario 1 represent a limited selection of clinically relevant variants, so our findings serve as a proof-of-concept that requires validation in larger, more diverse cohorts and real clinical specimens. This consideration is especially pertinent for the low-risk zone in Scenario 1, where the 0% false-negative rate was derived from only these 20 loci. Therefore, the absence of observed false negatives in this restricted dataset should not be interpreted as evidence of zero risk, and laboratory reports should acknowledge a residual false-negative risk.

### 4.6. Clinical Reference Framework

**Figure 2:**
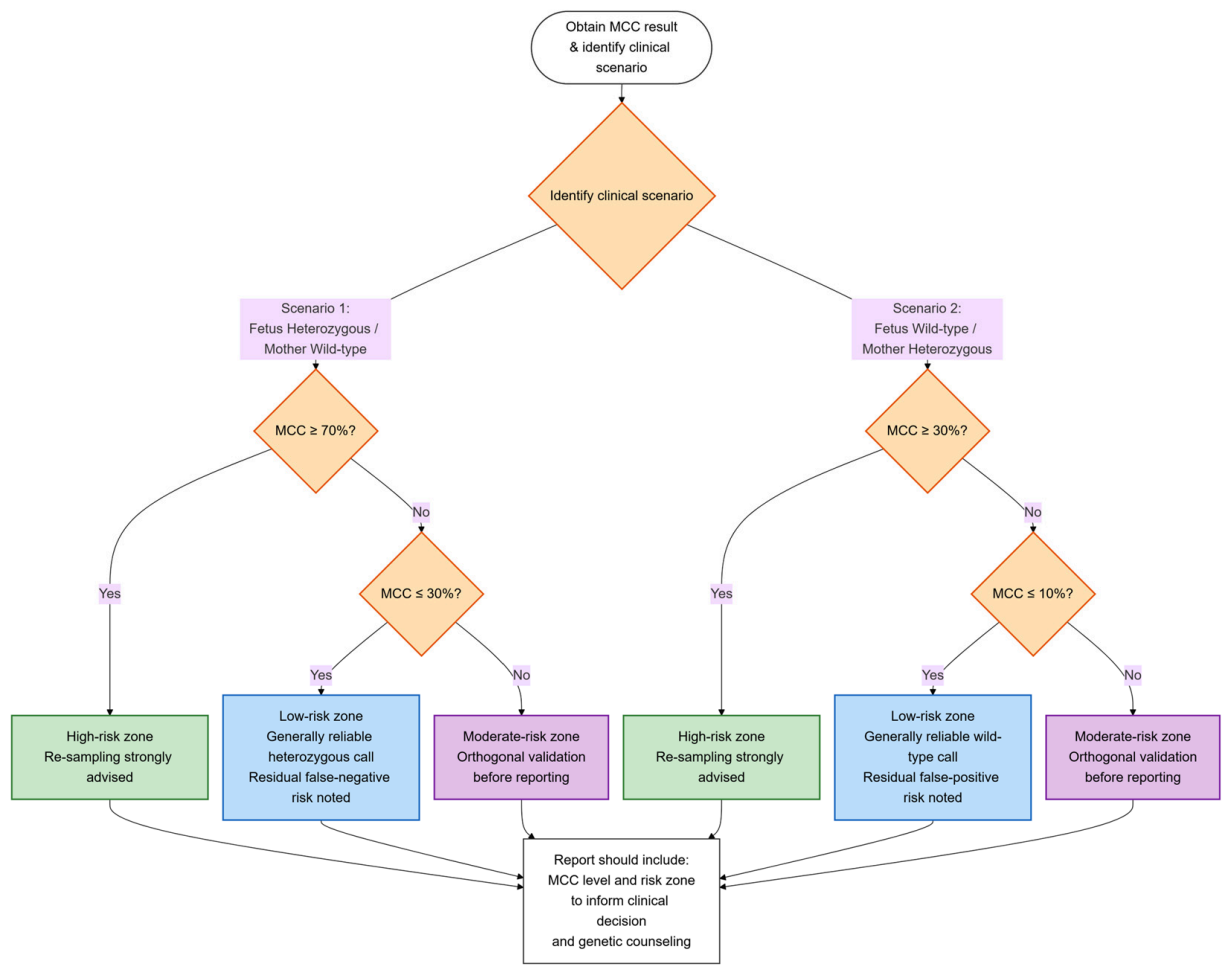
Flowchart of the clinical reference framework based on three-tier risk stratification.

Based on the three-tier risk stratification defined in Table 3, we propose the following reference framework for prenatal single-gene testing (Figure 2): (1) MCC levels should be obtained for all prenatal molecular diagnostic samples, and the result should be interpreted in conjunction with the clinical scenario. (2) If MCC falls within the high-risk zone (MCC ≥ 70% in Scenario 1, or MCC ≥ 30% in Scenario 2), re-sampling should be strongly considered. For MCC below these respective boundaries, the interpretation depends on the specific scenario—in Scenario 1, MCC in the low-risk zone (MCC ≤ 30%) generally supports a reliable heterozygous call, although a residual false-negative risk remains; MCC in the moderate-risk zone (30% < MCC < 70%) warrants orthogonal validation before reporting. In Scenario 2, MCC in the low-risk zone (MCC ≤ 10%) generally supports a reliable wild-type call, albeit with a residual false-positive risk; MCC in the moderate-risk zone (10% < MCC < 30%) also requires orthogonal validation before reporting. (3) The diagnostic report should include the MCC assessment results and the corresponding risk zone to support clinical decision-making and genetic counseling.

## 5. Conclusions

In conclusion, this study provides empirical boundaries for MCC tolerance in WES and Sanger sequencing, revealing a pronounced directional asymmetry between the two clinical scenarios and a three-tier classification of low-risk, moderate-risk, and high-risk zones. In Scenario 1 (fetus heterozygous/mother wild-type), the low-risk zone extends to 30% MCC, while the high-risk zone begins at 70% MCC. In Scenario 2 (fetus wild-type/mother heterozygous), the low-risk zone extends only to 10% MCC, and the high-risk zone starts at 30% MCC. Based on these findings, we further propose a clinical reference framework for interpreting sequencing results and guiding laboratory decisions, including reliable reporting, orthogonal validation, or re-sampling.

## Figures and Tables

**Table 1:** WES-based VAF quantification and false-negative/false-positive rates in Scenario 1 (Fetus Heterozygous/Mother Wild-type) and Scenario 2 (Fetus Wild-type/Mother Heterozygous).

MCC (%)	Scenario 1					Scenario 2				
	SNV		InDel		Total	SNV		InDel		Total
	Median VAF (Range) (%)	False-Negative Rate (%)	Median VAF (Range) (%)	False-Negative Rate (%)	False-Negative Rate (%)	Median VAF (Range) (%)	False-Positive Rate (%)	Median VAF (Range) (%)	False-Positive Rate (%)	False-Positive Rate (%)
5	50.3 (43.1–56.9)	0 (0/10)	40.2 (34.0–48.3)	0 (0/10)	0 (0/10)	0 (0–0)	0 (0/14)	0 (0–9.0)	0 (0/6)	0 (0/20)
10	43.7 (26.3–46.2)	0 (0/10)	38.1 (27.4–48.2)	0 (0/10)	0 (0/10)	0 (0–12.5)	0 (0/14)	3.8 (0–11.5)	0 (0/6)	0 (0/20)
15	39.0 (28.9–42.4)	0 (0/10)	37.5 (17.4–40.5)	0 (0/10)	0 (0/10)	10.8 (0–14.5)	0 (0/14)	12.8 (7.5–23.8)	33.3 (2/6)	10 (2/20)
20	33.2 (29.0–40.4)	0 (0/10)	31.6 (20.7–51.6)	0 (0/10)	0 (0/10)	15.2 (9.8–20.7)	57.1 (8/14)	13.0 (0–20.5)	33.3 (2/6)	50 (10/20)
30	27.1 (20.5–51.3)	0 (0/10)	26.7 (18.1–33.3)	0 (0/10)	0 (0/10)	18.6 (12.7–27.7)	71.4 (10/14)	19.9 (13.7–30.0)	66.7 (4/6)	70 (14/20)
50	23.6 (19.7–32.4)	0 (0/10)	25.8 (13.6–52.6)	10 (1/10)	5 (1/20)	21.1 (12.0–31.7)	92.9 (13/14)	22.6 (21.0–31.1)	100 (6/6)	95 (19/20)
60	18.7 (11.5–30.9)	20 (2/10)	20.0 (12.5–24.3)	20 (2/10)	20 (4/20)	28.8 (15.1–36.3)	100 (14/14)	33.0 (16.1–39.3)	100 (6/6)	100 (20/20)
70	13.4 (0–22.3)	60 (6/10)	12.2 (0–17.0)	80 (8/10)	70 (14/20)	33.7 (26.6–43.0)	100 (14/14)	35.0 (28.4–44.4)	100 (6/6)	100 (20/20)
80	10.0 (0–14.2)	100 (10/10)	1.1 (0–15.5)	90 (9/10)	95 (19/20)	36.4 (31.6–45.7)	100 (14/14)	38.8 (28.4–42.7)	100 (6/6)	100 (20/20)
90	0 (0–17.9)	90 (9/10)	0 (0–0)	100 (10/10)	95 (19/20)	45.0 (35.7–57.5)	100 (14/14)	41.3 (29.2–44.9)	100 (6/6)	100 (20/20)
95	0 (0–0)	100 (10/10)	0 (0–0)	100 (10/10)	100 (20/20)	44.6 (38.0–50.8)	100 (14/14)	46.6 (31.6–54.6)	100 (6/6)	100 (20/20)

Footnote: MCC, maternal cell contamination; WES, whole exome sequencing; VAF, variant allele frequency; SNV, single-nucleotide variant; InDel, insertions/deletions.

**Table 2:** True- and false-positive rates for SNVs and InDels across MCC gradients in Scenario 2 by WES.

MCC (%)	SNV (*n* = 11,743)		InDel (*n* = 884)		Total False-Positive Rate (%)
	True-Positive Rate (%)	False-Positive Rate (%)	True-Positive Rate (%)	False-Positive Rate (%)	
5	99.9	0.1	98.3	1.7	0.2
10	99.1	0.9	97.2	2.8	1.0
15	87.6	12.4	84.7	15.3	12.6
20	59.3	40.7	64.0	36.0	40.4
30	22.6	77.4	34.0	66.0	76.6
50	9.3	90.7	21.6	78.4	89.8

**Table 3:** Three-tier risk stratification for MCC in prenatal single-gene testing in two scenarios.

Scenario	Low-Risk Reference Zone	Moderate-Risk Reference Zone	High-Risk Reference Zone
Fetus Heterozygous/Mother Wild-type	≤30% MCC	30% < MCC < 70%	MCC ≥ 70%
Fetus Wild-type/Mother Heterozygous	≤10% MCC	10% < MCC < 30%	MCC ≥ 30%

Footnote: Low-risk zone: generally reliable detection with a residual false-negative or false-positive risk; moderate-risk zone: elevated risk of false negatives or false positives; high-risk zone: false-negative or false-positive rates exceeding 50%. The empirical boundaries were derived from simulated DNA mixing data from this study and require local validation before clinical adoption.

## Data Availability

The data presented in this study are available on request from the corresponding authors due to institutional restrictions.

## Supplementary Materials

The following supporting information can be downloaded at https://www.mdpi.com/article/10.3390/diagnostics16152302/s1, Table S1. Characteristics of the 40 loci analyzed in the two clinical scenarios. Table S2: Interference of maternal cell contamination on WES-based VAF quantification in two scenarios. Table S3. Mann–Whitney U test results on the VAF distributions between SNVs and InDels at each MCC gradient in two scenarios. Table S4: McNemar’s test results for paired comparisons between WES and Sanger sequencing across MCC gradients in two scenarios.

## Abbreviations

The following abbreviations are used in this manuscript:

MCC	Maternal cell contamination
WES	Whole exome sequencing
SNVs	Single-nucleotide variants
InDels	Insertions/deletions
CVS	Chorionic villus sampling
SNP	Single-nucleotide polymorphism
STR	Short tandem repeat
VAFs	Variant allele frequencies
